# A multi-modal open dataset for mental-disorder analysis

**DOI:** 10.1038/s41597-022-01211-x

**Published:** 2022-04-19

**Authors:** Hanshu Cai, Zhenqin Yuan, Yiwen Gao, Shuting Sun, Na Li, Fuze Tian, Han Xiao, Jianxiu Li, Zhengwu Yang, Xiaowei Li, Qinglin Zhao, Zhenyu Liu, Zhijun Yao, Minqiang Yang, Hong Peng, Jing Zhu, Xiaowei Zhang, Guoping Gao, Fang Zheng, Rui Li, Zhihua Guo, Rong Ma, Jing Yang, Lan Zhang, Xiping Hu, Yumin Li, Bin Hu

**Affiliations:** 1 Gansu Provincial Key Laboratory of Wearable Computing, School of Information Science and Engineering, Lanzhou University, Lanzhou, China; 2 CAS Center for Excellence in Brain Science and Intelligence Technology, Shanghai Institutes for Biological Sciences, Chinese Academy of Sciences, Shanghai, China; 3 Joint Research Center for Cognitive Neurosensor Technology of Lanzhou University & Institute of Semiconductors, Chinese Academy of Sciences, Lanzhou, China; 4 Open Source Software and Real-Time System (Lanzhou University), Ministry of Education, Lanzhou, China; 5 Shenzhen Institutes of Advanced Technology, Chinese Academy of Sciences, Shenzhen, China; 6 Lanzhou University Second Hospital, Lanzhou, China

**Keywords:** Diagnostic markers, Predictive markers, Reading, Depression

## Abstract

According to the WHO, the number of mental disorder patients, especially depression patients, has overgrown and become a leading contributor to the global burden of disease. With the rising of tools such as artificial intelligence, using physiological data to explore new possible physiological indicators of mental disorder and creating new applications for mental disorder diagnosis has become a new research hot topic. We present a multi-modal open dataset for mental-disorder analysis. The dataset includes EEG and recordings of spoken language data from clinically depressed patients and matching normal controls, who were carefully diagnosed and selected by professional psychiatrists in hospitals. The EEG dataset includes data collected using a traditional 128-electrodes mounted elastic cap and a wearable 3-electrode EEG collector for pervasive computing applications. The 128-electrodes EEG signals of 53 participants were recorded as both in resting state and while doing the Dot probe tasks; the 3-electrode EEG signals of 55 participants were recorded in resting-state; the audio data of 52 participants were recorded during interviewing, reading, and picture description.

## Abstract (summary)

**Table:** 

Measurement(s)	Human Brainwave • spoken language
Technology Type(s)	EEG collector • audio recorder
Sample Characteristic - Organism	Homo Sapiens
Sample Characteristic - Location	China

## Background & Summary

For the past decades, the number of mental disorder patients, especially depression patients, has overgrown. According to the WHO 2015 statistics, the total estimated number of global diagnosed depression patients reached 322 million^1^ and increased by 18.4% between 2005 and 2015^2^. Thus, major depressive disorder (MDD) has become a leading contributor to the global burden of disease. However, diagnosis of depression is currently based on interviews, and clinical scales carried out by professionals, such as psychiatrists and psychologists. The process is not only labor-consuming but also time-consuming. The result of depression diagnosis is also not as convincing as some other illnesses, such as hypertension, due to its lack of physiological indicators. These reasons are causing the global population to still be widely untreated for their mental health disorders.

As non-invasive physiological data, Electroencephalography (EEG) provides a direct measure of postsynaptic potentials with millisecond temporal resolution. EEG trades for a higher temporal resolution at the cost of low spatial resolution. Acharya *et al*.^3^ proposed a technique that can learn automatically from the input EEG signals to differentiate EEGs obtained from depressive and normal participants. And it was discovered that the EEG signals from the right hemisphere are more distinctive in depression than those from the left hemisphere. Allen *et al*.^4^ found frontal EEG asymmetry may serve as a biomarker of depression risk. Tement *et al*.^5^ focused on analyzing EEG alpha frequency and suggested burnout was associated with alpha power, whereas depression was linked to individual alpha frequency. Whitton *et al*.^6^ used high temporal resolution of EEG to compare the spectral properties of resting-state functional connectivity in individuals with a major depressive disorder to healthy controls. Their research discovered that elevations in high-frequency default mode networks and the frontoparietal network connectivity might be a neural marker linked to a more recurrent illness course.

Recordings of spoken language are another non-invasive accessible physiological data. Studies have shown that mental disorders will be causing the patients’ recorded speech data to differ from healthy controls. Harati *et al*.^7^ built their predictive model on top of emotion-based features to help clinical management decisions during Deep Brain Stimulation treatment of major depressive disorder patients. Cummins *et al*.^8^ studied speech-based depression classification using gender dependant features and classifiers and revealed gender differences in the effect of depression on vowel-level formant features. Williamson *et al*.^9^ proposed an algorithm that estimates the articulatory coordination of speech from recordings of spoken language and uses these coordination features to learn a prediction model to track depression severity.

For researchers in the field, good-quality data is essential for their analysis results. However, good quality EEG and recordings of spoken language data are hard to be acquired. Firstly, experiment participants have to be adequately diagnosed by professional doctors, not by self-rating scales. The reason is that although some well-recognized self-rating scales are suitable for self-evaluations, they are not as comprehensive as clinically diagnosed. Secondly, the experiment has to be conducted, and data has to be collected before patients taking any medication since the medication will cause brain activity to change drastically. Last and most important, the experiment requires full cooperation from the participants, who are already depressed. One of the symptoms of major depressive disorder patients is the lack of motivation to do anything. Therefore, it is tough to ask patients to cooperate through the whole experiment process, which could last hours.

Here we present a multi-model open dataset for mental-disorder analysis. For now, the dataset includes data mainly from clinically depressed patients and matching normal controls. At this stage, only electroencephalogram (EEG) and recordings of spoken language recording data are made publicly available. The EEG dataset includes data collected using a traditional 128-electrodes mounted elastic cap and a novel wearable 3-electrode EEG collector for pervasive computing applications. The 128-electrodes EEG signals of 53 participants were recorded as both in resting state and while doing the Dot probe tasks; the 3-electrode EEG signals of 55 participants were recorded in resting-state; the recordings of spoken language data of 52 participants were recorded during interviewing, reading, and picture description. We encourage other researchers in the field to use it for testing their methods of mental-disorder analysis.

The traditional 128-electrodes EEG data have been used in several studies^10–15^. Using the Dot probe tasks EEG, Li *et al*. suggested MDD patients show difficulty in attention disengagement from negative stimuli, reflected by P300. The CFS over other methods leads to a good overall performance in most cases, especially when the KNN classifier is used for P300 component classification, illustrating that the ERP component may be applied as a tool for auxiliary diagnosis of depression^10^. Hu *et al*. suggested that a negatively sad emotion influenced cognitive attentional control in MDD in the early and late attentional stages of cognition. P200 and P300 might predict potential neurocognitive mechanisms underlying the dysregulated attentional control of MDD^11^. Using the resting-state EEG, Sun *et al*. showed that the functional connectivity feature PLI is superior to linear and nonlinear features. And when combining all the types of features to classify MDD patients, we can obtain the highest classification accuracy 82.31% using the ReliefF feature selection method and logistic regression (LR) classifier^12^. Sun *et al*. found that the combination of the imaginary part of coherence and cluster-span threshold outperformed other methods. Based on this combination, right hemisphere function deficiency, symmetry breaking, and randomized network structure were found in MDD, which confirmed that MDD had aberrant cognitive processing. Furthermore, the clustering coefficient in the left central region in the theta band and node betweenness centrality in the right temporal region in the alpha band was significantly negatively correlated with depressive level. And these network metrics could discriminate MDD from NC^13^. Peng *et al*. and Li *et al*. suggested a stronger brain interaction in the MDD group and a left-right functional imbalance in the frontal regions for MDD controls^14,15^. The recordings of spoken language data have been used in Liu’s study. Liu *et al*. proposed a new speech segment fusion method based on decision tree to improve the depression recognition accuracy. The recognition accuracy is 75.8% and 68.5% for males and females, respectively, on gender-dependent models^16^. The 3-electrode EEG data has been used in several studies^17–19^. Shi *et al*. showed three-channel resting-state EEG and features could distinguish the subjects between depression and normal beings; the classification accuracy is 72.25%^17^. Cai *et al*. used three-channel resting-state EEG compared with the EEG under audio stimulation and fused using a feature-level fusion technique to construct a depression recognition model. The highest classification accuracy of 86.98% was obtained^18,19^.

## Methods

Written informed consent was obtained from all participants prior to the experiment. The local Ethics Committee approved consent forms and study design for Biomedical Research at the Lanzhou University Second Hospital according to the World Medical Association (Declaration of Helsinki). These methods are expanded versions of descriptions in our related work^11,13,20,21^. All the experiments were performed separately.

### Participants

#### Full brain 128-electrodes EEG experiment

53 participants include a total of 24 outpatients (13 males and 11 females; 16–56-year-old) diagnosed with depression, as well as 29 healthy controls (20 males and 9 females; 18–55-year-old) were recruited;

#### 3-electrodes EEG experiment

55 participants include a total of 26 outpatients (15 males and 11 females; 16–56-year-old) diagnosed with depression, as well as 29 healthy controls (19 males and 10 females; 18–55-year-old) were recruited;

#### Recordings of spoken language experiment

52 participants include a total of 23 outpatients (16 males and 7 females; 16–56-year-old) diagnosed with depression, as well as 29 healthy controls (20 males and 9 females; 18–55-year-old) were recruited.

All participants had a normal or corrected-to-normal vision. Patients with major depressive disorder (MDD) were recruited among inpatients and outpatients from Lanzhou University Second Hospital, Gansu, China, diagnosed and recommended by at least one clinical psychiatrist. Posters recruited the normal controls (NC). The study was approved by the Lanzhou University Second Hospital Ethics Committee, and written informed consent was obtained from all participants before the experiment began. All MDD patients received a structured Mini-International Neuropsychiatric Interview (MINI)^22^ that met the diagnostic criteria for major depression of the Diagnostic and Statistical Manual of Mental Disorders (DSM) based on the DSM-IV^23^. The inclusion criteria for all participants were the age should between 18 and 55 years old and primary or higher education level. For MDD patients, the inclusion criteria were the diagnostic criteria of MINI met the criteria for depression, the Patient Health Questionnaire-9item (PHQ-9)^24^ score of participants was more significant than or equal to 5, and no psychotropic drug treatment having been performed in the last two weeks. For MDD patients, the exclusion criteria were having mental disorders or brain organ damage, having a severe physical illness, and extreme suicidal tendencies. For NC, the exclusion criteria included a personal or family history of mental disorders. In addition, the exclusion criteria for all participants were abused or dependent on alcohol or psychotropic drugs in the past year, pregnant women and lactation, or taking birth control pills. Each participant received a bonus compensation of approximately USD $16 for participating in this experiment.

### Experimental material

#### Full brain 128-electrodes EEG experiment

Task 1: Resting state

No experimental material. The participants should keep quiet and close their eyes as much as possible.

Task 2: Dot probe

The dataset we present comprises facial pictures from the standardized native Chinese Facial Affective Picture System (CFAPS)^25^. The facial images were chosen and classified into four sets as fear, sad, happy, and neutral emotion based on their valence. Two different valences of facial images (one belonging to emotional groups, the other to neutral groups) were selected arbitrarily. The stimuli pairs consisting of emotional and neutral facial pictures appeared side by side on the screen. The distance between the two facial images was 12 cm, with a constant viewing angle of 14.25°. Thus, we obtained 60 emotional-neutral face pairs, including 20 fear-neutral, 20 sad-neutral, and 20 happy-neural faces. The number of male and female pictures of each emotion was equal. The picture size was 5.16 cm × 5.95 cm, and all non-facial features were trimmed (i.e., hair or clothing). The MATLAB software was used to equate mean pixel luminance, contrast, and Centro-spatial frequency of all face pictures, and the pictures were converted into 8-bit greyscale images.

#### 3-electrodes EEG experiment

Only resting-state EEG was recorded; therefore, there is no experimental material used. However, the participants should keep quiet and close their eyes as much as possible.

#### Recordings of spoken language experiment

In the design of the experiment, two factors were examined: speaking style and emotional valence. Speaking style is about three speaking patterns involved in the study: interview, word reading, and picture description. Each of them had three kinds of emotional valences: positive, neutral, and negative. The order of speech with different emotional valences was assigned randomly to counteract the sequence effect. The language of the experiment was Chinese and the whole experiment lasts about 25 minutes.

More details of the three speaking styles are described as follows:

1. The interview had 18 questions that came from DSM-IV^23^, HRSD^26^, and other scales.
2. Reading comprised two groups of words, and each group has ten common Chinese words that came from affective ontology corpus^27^ and Chinese sentimental words extremum table^28^.
3. The picture description contained three facial expression pictures, which were from the Chinese Facial Affective Picture System (CFAPS)^29^. More details for experimental procedures could be found in^30^.

### Experimental equipment

#### Full brain 128-electrodes EEG experiment

Continuous EEG signals were recorded using a 128-channel HydroCel Geodesic Sensor Net (Electrical Geodesics Inc., Oregon Eugene, USA) and Net Station acquisition software (version 4.5.4). The sampling frequency was 250 Hz. All the raw electrode signals were referenced to the Cz. For each participant, we first measured their head circumference and then selected the appropriate size EEG net. The impedance of each electrode was checked before recording to ensure good contact and was kept below 50 kΩ^26^.

#### 3-electrodes EEG experiment

**Fig. 1:**
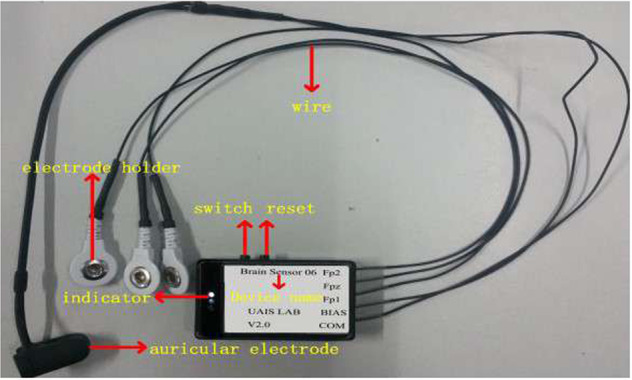
Three-electrode pervasive EEG collection device.

Data was recorded by a 24-bit A / D converter record EEG signals at a sampling frequency of 250 Hz, as shown in Fig. 1.

#### Recordings of spoken language experiment

The experiments were performed in a quiet, clean, soundproof, and no electromagnetic interference room. During the experiment, the ambient noise of the lab must be less than 60 dB. The devices we used for recording are Neumann TLM102 (microphones) and RME FIREFACE UCX (audio card), with a 44.1 kHz sampling rate and 24-bit sampling depth. All recording data were saved in uncompressed WAV format.

The whole experiment lasted about 25 minutes for one participant. During recording, the participant was asked not to touch any equipment and keep the distance between mouth and microphone about 20 cm. Each participant is invited to complete all three experimental tasks on a comfortable chair. Ambient noise signals were required under 60 dB to prevent interference with the participant’s audio signals.

### Experimental paradigm

#### Full brain 128-electrodes EEG experiment

The multi-channel EEG was collected in a quiet, soundproof, well-ventilated room without solid electromagnetic interference. Participants completed the tasks sitting alone in the room while the operators monitored their progress in the adjoining room. When the electrode placement is completed, and the impedance meets the requirements, data acquisition can be started. All participants were asked to complete two tasks: resting state and dot-probe tasks.

Task 1: Resting state

5 minutes of eyes-closed resting-state EEG was recorded. Participants were required to keep awake and still without any bodily movements, including heads or legs, and any unnecessary eye movements, saccades, and blinks^31,32^. After completion of Task 1, participants had a rest and then completed Task 2.

Task 2: Dot probe

Participants were seated in front of the monitor (17” monitor, 1280 × 1024 resolution, and 60 Hz refresh rate) at a distance of 60 cm. All relevant instructions were shown on the computer screen initially. Before the experiment began, the participants were instructed to complete the 10 practice trials to get familiar with the task. The participants were asked to focus their attention on the emotional-neutral face pairs with eyes viewing freely in the formal experiment. And they were asked to press the button on the reaction box as quickly and accurately as possible when the dot appeared. The participants must press down the controller without any bodily movements, including heads or legs, and any unnecessary eye movements, saccades, and blinks. After completing each block, they would have a rest^33^.

**Fig. 2:**
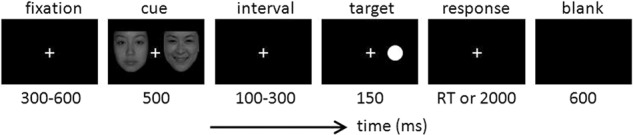
The trial sequence of the dot-probe task. The cue stimuli include three kinds of emotional-neutral face pairs (Fear-Neutral, Sad-Neutral, and Happy-Neutral). The dot target was presented randomly as a target in either the left or right position of the fixed cross.

The whole experimental paradigm was programmed by E-prime v2.0 (Psychology Software Tools, Inc., Pittsburgh, PA, USA). The task consisted of three blocks (Fear-Neutral, Sad-Neutral, and Happy-Neutral), and each block had 160 trials. At the beginning of each trial, a fixed white cross appeared on the central screen at 300 ms and lasted for 300 ms from the start. Then, the cross was presented on the screen centrally on the screen throughout the experiment. The emotional-neutral face stimuli pair was shown on the screen as a cue for 500 ms; the pair was arranged in a pseudo-random order. After a short interval from 100–300 ms, the dot-probe appeared randomly as a target on the left or right position of the fixed cross for 150 ms. Concurrently, the participant was asked to identify the spatial location of the ‘dot’ and to record their response by pressing the button ‘1’ or ‘4’ on the reaction box with their index fingers as quickly as possible. If the dot appeared to the left of the fixation cross, the participant should press ‘1’; if the dot appeared to the right of the fixation cross, the participant should press ‘4’. An automatic interval of 2000 ms was used to receive the participant’s response; otherwise, the participant would be directed into the subsequent trial followed by a black screen presented for 600 ms. The procedure continued gradually until a block was completed. Each block was also run in a cycle manner until the entire task was finished. The whole experimental task was completed in about 25 minutes. The trial sequence of the dot-probe task is illustrated in Fig. 2.

#### 3-electrodes EEG experiment

The data were recorded in a room without loud noise and strongly magnetic. Participants kept their eyes closed until they were observed their EEG signals were relatively stable, then we started a 90-second data acquisition.

#### Recordings of spoken language experiment

There are three fixed-order parts: interview, reading, and picture description. Finally, the text materials were shown on a computer screen, and the participants were asked to finish the experiment following the instructions.

1. Interview: This task contained 18 questions with positive, neutral, and negative meanings. These topics came from DSM-IV and some depression scales, which are often used in this field. For example: If you have a vacation, please describe your travel plans^30^. What is the best gift you have ever received, and how did you feel^34^? Please describe one of your friends, including age, job, characters, and hobbies. How do you evaluate yourself? What would you like to do when you are unable to fall asleep? What makes you desperate?
2. Reading: This part consists of a short story named “The North Wind and the Sun”, which is from the booklet “The Principles of the International Phonetic Association”^35^, and often used in the acoustic analysis in international, multilingual clinical research. And three groups of words with positive (e.g., outstanding, happy), neutral (e.g., center, since), and negative (e.g., depression, wail) emotion. Positive and negative words are selected from the affective ontology corpus created by Hongfei Lin (http://ir.dlut.edu.cn/Group.aspx?ID = 4) and neutral ones are picked out from the Chinese affective words extremum table (http://www.datatang.com/data/43216). These words are often used in Chinese to avoid educational level, and three groups of words have close stroke numbers. Participants are told to read a story and these words in their ordinary ways.
3. Picture description: The materials for this task include four pictures in all. Three pictures, which express positive, neutral, and negative faces, are selected from the Chinese Facial Affective Picture System (CFAPS), and the last one with a “crying woman” came from Thematic Apperception Test (TAT)^34^. Murray creates TAT in 1935, which is used in psychological counseling and psychotherapy at present. In this task, participants are told to describe these four pictures freely.

## Data Records

Datasets described in this paper have been made available as safeguarded data on the UK Data Archive’s data repository ReShare^36^. Anyone wishing to download and use these data must register with the UKDA and agree to their End User Licence conditions, outlined at https://ukdataservice.ac.uk/cd137-enduserlicence/. For commercial use, please contact the UK Data Service at help@ukdataservice.ac.uk.

The data collection includes three main data parts: Full brain 128-electrodes EEG data, 3-channel resting-state EEG data, and recordings of spoken language. Full brain 128-electrodes EEG data are stored in two compressed files, ‘EEG_128channels_resting_lanzhou_2015.zip’ for resting-state recordings; and ‘EEG_128channels_ERP_lanzhou_2015.zip’ for Event-Related Potential recordings. 3-channel resting-state EEG data are stored in one package, which is named ‘EEG_3channels_resting_lanzhou_2015.zip’. The recordings of spoken language are also stored in one package as ‘audio_lanzhou_2015.zip’. 128-electrodes EEG data are also available in Brain Imaging Data Structure (BIDS) format, which is compressed in ‘MODMA EEG_BIDS_format.zip’. Behavioral data of all the participants are available in package ‘Behavioral_Data.zip’. A translated copy of the original informed Consent and a sample of the Information Sheet used in each data packages are also provided as ‘InformedConsent.pdf’ and ‘InformationSheet.pdf’, respectively.

The raw experimental dataset can also be downloaded from the publicly accessible repository free of charge at http://modma.lzu.edu.cn/data/index/. All the users interested in this dataset will need to sign an End User License Agreement (EULA) before their downloads. The raw datasets are packaged under “EEG” and “Recordings of spoken language” categories. Within each package, there is an excel file containing demographic data and psychological assessment scores of all the participants of that dataset. Each experiment participant is given an identity number, and this participant id is unique across all the packages. In the future, more data will be added regularly, which will cover not only more mental disorders, such as schizophrenia, anxiety, and mania but also more data types, such as eye-movement tracking, facial expression recording, and MRIs. We encourage other researchers in the field to use it for testing their methods of mental-disorder analysis.

### Data recording and storage

#### Full brain 128-electrodes EEG experiment

Task 1: Resting state

Five minutes of eyes-closed resting data were recorded with Net Station acquisition software. First, the acquired raw data were saved as .mff files on the MAC PC. The data files named with the “0201” prefix represent data from patients with MDD, and the data files called with the “0203” prefix represent data from NC. Then .mff files were converted to.mat files using the Net Station Waveform Tools. Then .mff files were converted to .mat files using the Net Station Waveform Tools and were stored in the/sourcedata directory. To comply with the BIDS format^37^, the .mat format was further converted to .EDF format, which was stored in the/eeg directory of each participant. Generally, you can find data in the .tsv files and descriptions in the accompanying .json files. In addition, the relationship between the original participants id (e.g., 0201/0203XX) and BIDS participants id (e.g., sub-YY) were described in the sheet1 of participants_information_EEG_128channels_resting_lanzhou_2015.xlsx.

Task 2: Dot probe

The stimulus computer presented the dot-probe experiment task during the experiment and recorded the reaction time(RT), accuracy, and CellNumber in a .edat file. The data files named with the “Dot_Detection-0201” prefix represent data from patients with MDD, and the data files called with the” Dot_Detection-0203” prefix represent data from NC. Thus, E-prime can improve the event file. The stimulus computer also sent synchronized triggers to the Net Station acquisition software. Concurrently, the Net Station acquisition software recorded EEG data with the timestamps of triggers. The acquired raw data were saved as .mff files on the MAC PC. The data files named with the “0201” prefix represent data from patients with MDD, and the data files called with the “0203” prefix represent data from NC. The original data converted to .raw files using the Net Station Waveform Tools, and the original data and behavioral data were stored in the/sourcedata directory. Then, the.raw format was further converted to .EDF format, the .EDF files and events .tsv files were held in the/eeg directory of each participant. In addition, the .edat files are stores in the/behavioral data directory for each participant. Likewise, the relationship between the original participants id (e.g., 0201/0203XX) and BIDS participants id (e.g., sub-YY) were described in the sheet1 of participants_information_EEG_128channels_ERP_lanzhou_2015.xlsx.

For the two experimental tasks, EEG signals were obtained using the HydroCel Geodesic Sensor Net (HCGSN) (Electrical Geodesics Inc., Oregon Eugene, USA). Recorded EEG signals were collected using a wired EEG cap with 128-Ag/AgCl electrodes. The contact impedance between all electrodes and the skin was kept below 50 kΩ. The EEG recordings were amplified by the Electrical Geodesics amplifiers and digitized at 250 Hz. Net Station acquisition software 4.5.4 is the ultimate tool for data acquisition.

The data are stored in folders by task, such as “EEG LZU_2015_2_resting state” and “EEG LZU_2015_2_dot probe”. One file per participant. The behavioral data are also included in the .edf files.

#### 3-electrodes EEG experiment

**Fig. 3:**
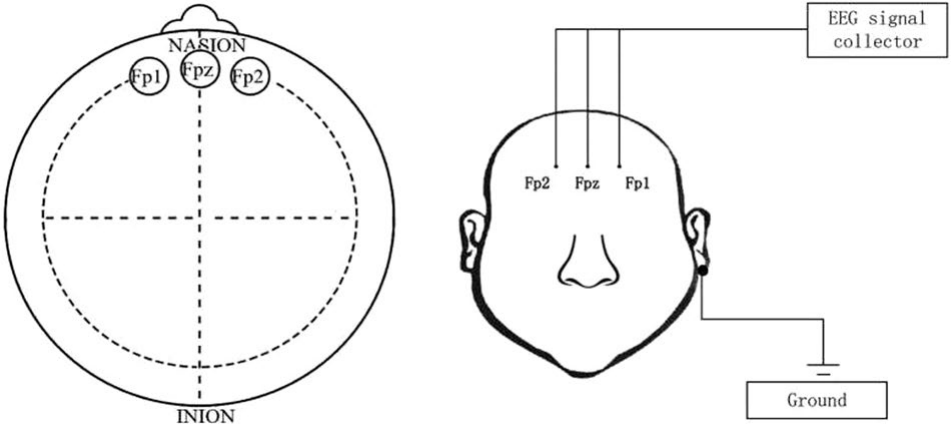
Location of the three electrodes placement and the three-electrode pervasive EEG collection device.

Considering that the prefrontal lobe strongly correlates with emotional processes and psychiatric disorders, we collected the EEG signal by three-electrode pervasive EEG collection device with three electrodes located on the prefrontal lobe (Fp1, Fpz, and Fp2). The location of the three electrodes placement (Fp1, Fpz, and Fp2) and the three-electrode pervasive EEG collection device are shown in Fig. 3.

Collected data files were stored in a referential montage using an open-source TXT format. Each TXT is an M by N array, M is the number of electrodes (M = 8), and N is the number of all sample dots. What needs to be explained here is the first three electrodes correspond to the Fp1 electrode, Fpz electrode, Fp2 electrode, and the last five electrodes are alternate channel data, which is the default value if not be used.

#### Recordings of spoken language experiment

We collected data in a quiet, clean, soundproof, and no electromagnetic interference room. During the experiment, the ambient noise of the lab must be less than 60 dB. The devices we used for recording are Neumann TLM102 (microphones) and RME FIREFACE UCX (audio card), with a 44.1 kHz sampling rate and 24-bit sampling depth. All recording data were saved in uncompressed WAV format.

All the recordings were segmented and labeled manually, and only participants’ speech was kept. Thus, there were 29 recordings (interview (18), passage reading (1), word reading (6), and picture description (4)) for each participant.

### 3-electrodes EEG signals

**Fig. 4:**
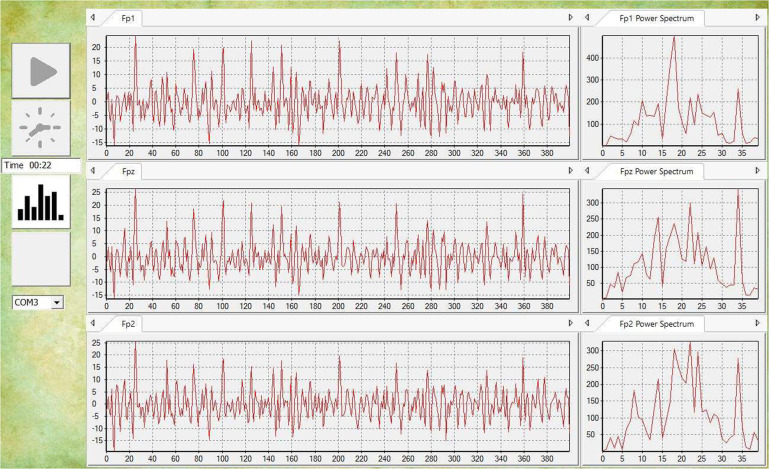
The real-time visual display system shows the EEG signals after processing in real-time.

During processing, we converted the raw hex data of the first three columns to decimal data first. Then, the signal was filtered by a 1 Hz high-pass and 45 Hz low-pass finite impulse response (FIR) filter. Next, we use an adaptive noise canceller to remove the eye-blink artifacts. Figure 4 shows the signal waveform in real-time after preprocessing.

### Whole-brain EEG signals

Task 1: Resting state

The raw files were read using the EEGLAB toolbox in MATLAB. The uploaded files named with mat/EDF suffixes contain all the signals. After loading the files, the “EEG.data” variable included 129 EEG signals. The first 128 signals were from electrode E1 to electrode E128. The last signal from Cz was the reference electrode.

Task 2: Dot probe

The raw files were read using the EEGLAB toolbox in MATLAB. The uploaded files named with raw/EDF suffixes contain all the signals. After loading the files, the “EEG.data” variable included 128 EEG signals. The 128 signals were from electrode E1 to electrode E128. Additionally, as shown in Table 1, the types of events (see “EEG.event.type”) in the dataset were classified as fixation onset (mark: hfix or ffix or sfix), cue onset (mark: hcue or fcue or scue), interval onset (mark: hisi or fisi or sisi), target onset (mark: hdot or fdot or sdot) and response onset (mark: hwrp or fwrp or swrp).

**Table 1:** The timestamps of triggers of different experiment blocks in the dot-probe task.

Happy-Neutral block				
‘hfix’	‘hcue’	‘hisi’	‘hdot’	‘hwrp’
fixation onset	cue onset	interval onset	target onset	response onset
Fear-Neutral block				
‘ffix’	‘fcue’	‘fisi’	‘fdot’	‘fwrp’
fixation onset	cue onset	interval onset	target onset	response onset
Sad-Neutral block				
‘sfix’	‘scue’	‘sisi’	‘sdot’	‘swrp’
fixation onset	cue onset	interval onset	target onset	response onset

### Recordings of spoken language

In the experiment, 29 recordings for every single participant were stored and named as 1 to 29 in a determined sequence. The details were as follows: The positive, neutral, and negative interview recordings are called 1-6, 7-12, and 13-18 separately. The record of the short story is named 19. The six-word groups’ readings are called 20-21, 22-23 and 24-25 according to the sequence of positive, neutral, and negative emotion. 26-28 were the picture description with the same order as the reading part. The record of TAT was numbered 29.

## Technical Validation

### Behavioral validation

#### Full brain 128-electrodes EEG experiment

For both of the two tasks (Resting-state and Dot probe), the EEG dataset was collected from 24 participants with MDD and 29 NC. All participants had a normal or corrected-to-normal vision. Patients with MDD were recruited among inpatients and outpatients from Lanzhou University Second Hospital, Gansu, China. Posters recruited the NC. At the beginning of the experiment, each participant was given approximately 10 minutes to read the experiment prevalent instructions and fill in the participant information questionnaire. Next, the participant would wear a suitable cap and began to record the EEG signal.

For the dot-probe task, RT was defined as the time period between target onset and response onset. The trials would be rejected if RT is less than 100 ms or more than 2000 ms, and all of the trials in which the participants failed to respond would be excluded from the analyses. In addition, according to whether the dot in the target and the emotional face in the cue appear on the same side or the opposite side, we can divide the data into two conditions: emotional congruent and emotional incongruent. In this study, a total of 6 conditions were included: Happy-congruent, Happy-incongruent, Sad-congruent, Sad-incongruent, Fear-congruent, and Fear-incongruent. The corresponding relationship of conditions is shown in Table 2. Researchers can extract and analyze the EEG data under related stimuli according to the questions they are interested in. For instance, if someone is interested in the Happy-congruent condition, he can extract and analyze the corresponding EEG data according to the tag “1” of CellNumber in the .edat files.

**Table 2:** The CellNumber of different emotional-congruent/incongruent condition in dot-probe task.

CellNumber	Emotional-congruent/incongruent Condition
1	Happy-congruent condition
2	Happy-incongruent condition
3	Sad-congruent condition
4	Sad-incongruent condition
5	Fear-congruent condition
6	Fear-incongruent condition

EEG signals were recorded using 128-Ag/AgCl electrodes elastic Cap (HydroCel Geodesic Sensor Net, HCGSN). The electrode skin interface was prepared by cleaning and rubbing the skin and then applying KCL-based conductive gel. The impedance of the electrodes was calibrated repeatedly until below 50 kΩ. EEG signals from the electro-cap were amplified using the Electrical Geodesics amplifiers (Electrical Geodesics Inc., Oregon Eugene, USA) and recorded at a sampling rate of 250 Hz.

#### 3-electrodes EEG experiment

Professional psychologists assisted all participants in completing the Mini-Mental State Checklist(MMSE) as a preliminary judgment of depression. If the participant is at high risk of depression, a Simple Self-Test Depression Scale(PHQ-9) will be filled to judge the level of depression. All essential information is collected at the same time.

According to the information on the self-assessment questionnaire and the selection criteria, the candidate participants were judged whether they met the experimental criteria or not. All the participants washed their hair under the leadership of the staff, then wear experimental equipment in an excellent experimental environment.

It should be noted that all participants within the first two weeks of the experiment should not take any psychotropic drugs and do not have any other mental illnesses or brain organ damage (such as epilepsy). In addition, women with depression who are pregnant should be confirmed that: Women who are in lactation or taking birth control pills and who have been abused or depended on alcohol or psychotropic substances in the past one year should not participate in the experiment.

During the experiment, according to the international 10-20 system electrode placement standards, we choose 3 positions on the forehead to place the electrodes.

#### Recordings of spoken language experiment

All participants are asked to sign informed consent, and they all have a certain level of education, which means that they can understand the questions and answer accurately. In addition, before participating in the experiment, the participants were told to answer the question as realistically as possible, which makes these data authentic.

### 3-electrodes EEG signals validation

We selected FP2 and FP1 leads of EEG signals of the depression group (n = 11) and the control group (n = 11), each lead of the EEG data first through 0.5∼30 Hz of the FIR band-pass filter. Then, we calculate the relative power of δ(0.5∼4 Hz), θ(4∼8 Hz), α(8∼14 Hz) and β(14∼30 Hz) rhythms of two leads EEG FP2, FP1. Next, we used SPSS 19.0 software packages to do a paired sample T-test of the left and right brainαrhythm of two groups. Statistical analysis shows that a significant difference is found in the relative power of the left and right brain signals α rhythm of the depression group of the α rhythm, with statistical significance. In comparison, the relative power of the control group α rhythm does not reach a significant level, with no statistical significance.

For raw public data, we have all preprocessed and identified the data quality through experiment experience.

### Whole-brain EEG signals validation

EEG data of both tasks (Resting-state and Dot probe) were raw data, which were saved in the MODMA dataset.

### Recordings of spoken language validation

The experiments were performed in a quiet, clean, soundproof, and no electromagnetic interference room. Each recording was gathered following the basic process: one-minute rest, finishing the task showed on the computer screen, and another break for one minute. During the experiment, if the noise is more than 60db, it will not be recorded. Besides, our good recording equipment makes the signal ‘quality’ good.

The quality of the sound recordings is an informal and rough indication of the ratio of signal power to noise power (SNR). The SNR ranges from 20 to 30 and can be calculated typically.

## Data Availability

No custom code was used to generate or process the data described in the manuscript.
